# Kingsnorth's modified score as predictor of complications in open inguinal hernia repair

**DOI:** 10.1007/s13304-022-01341-2

**Published:** 2022-08-09

**Authors:** Alejandro Bravo-Salva, Margarita Salvá Puigserver, Clara Téllez-Marqués, Marc Pérez-Guitart, Alba González-Martín, J. J. Sancho-Insenser, M. Pera-Roman, José A. Pereira-Rodríguez

**Affiliations:** 1 Abdominal Wall Surgery Unit, Section of General Surgery, Department of General Surgery, Parc de Salut Mar, Hospital del Mar Medical Research Institute (IMIM), Passeig Maritim 25-29, 08003 Barcelona, Spain; 2 Department of Medicine and Life Sciences, Universitat Pompeu Fabra, Barcelona, Spain; 3 Department of General Surgery, Hospital Comarcal del Alt Penedés, Barcelona, Spain; 4 Surgery Department, Universitat Autónoma de Barcelona, Barcelona, Spain

**Keywords:** Unilateral inguinal hernia, Classification, Complications, Groin hernia repair, Hernia registries

## Abstract

**Purpose:**

This study aims to analyse the postoperative complications (30 days) on unilateral primary inguinal hernia repair and prove their correlation with the preoperative modified scoring system of Kingsnorth (KN).

**Methods:**

Prospective study design collecting data from patients who underwent surgery for unilateral primary inguinal hernia in a University Hospital. The data were collected in the National Inguinal Hernia Registry (EVEREG). A statistical analysis to assess the association between the presence of postoperative complications and the preoperative and intraoperative variables was performed. The patients were classified depending on their KN score. Surgical complications and their relationship with the classification were specifically analysed. Study design was performed following STROBE statements.

**Results:**

The sample included 403 patients who met the inclusion criteria from which 62 (15.3%) subjects presented postoperative complications. The variables that presented a statistically significant relationship with the appearance of complications were a KN score of 5–8 (OR 2.7; 95% CI 1.07–4.82; *P* = 0.03) and the involvement of a member of the abdominal wall surgery unit in the procedure (OR 0.28; 95% CI 0.08–0.92; *P* = 0.03). The KN score correlated with a longer duration of surgery (Pearson's correlation 0.291; *P* < 0.0001).

**Conclusion:**

The KN classification can predict the onset of surgical wound complications on patients who undergo a primary unilateral inguinal hernia surgery. A KN score of 5–8 has a higher probability of wound complications. When surgery is performed by the abdominal wall surgery unit, the chances of postoperative complications decrease.

## Introduction

Abdominal wall hernias are one of the most frequent surgical conditions. It is estimated that worldwide, around 20 million hernias are operated annually [1]. Inguinal hernias are the most frequent procedure and represent 75% of all abdominal wall hernias [2].

Throughout the history of surgery, numerous classifications of inguinal hernias have been proposed. The disadvantages of these classifications were that they were defined after surgery and complex. As a result, very simple anatomical localization systems were used (indirect, direct and femoral), which are not useful to predict technical difficulties, complications or recurrences [3].

Currently, the European Hernia Society (EHS) has proposed a simpler classification system based on operative anatomy and hernia size [4]. This system could be useful as a predictor, but it remains postoperative. It consists of a combination of letters and numbers according to hernia characteristics. The letter indicates the position, assigning the letter L to those in lateral position (indirect or external oblique hernias), the letter M to those medial to the inferior epigastric vessels (direct hernias) and the letter F to femoral hernias. The number corresponds to the size of the hernial defect. If it is smaller than 1.5 cm it corresponds to number 1, between 1.5 and 3 cm it corresponds to number [2] and if it is larger than 3 cm it is classified as 3. Finally, if the hernia is primary it would then be classified as P and if it is recurrent as R [4].

In summary, there are many classification systems, but they are heterogeneous and not very accurate to predict the evolution of the patient and the surgery [3].

In 2004, Kingsnorth et al. designed a preoperative classification system that considered hernia characteristics and scapular fold thickness [5] (Table 1). His work showed that this system could predict the difficulty of the surgical procedure. However, its correlation with the development of postoperative complications and recurrences has not been analysed.

**Table 1:** Original and modified Kingsnorth classification

Hernia characteristics	Patient characteristics original classification	Patient characteristics modified classification
H1: Groin only; reduces spontaneously when lying down	F1: SF < 15 mm	F1: BMI < 25
H2: Groin only; reduces completely with gentle manual pressure	F2: SF 15–24.9 mm	F2: BMI 25–29,9
H3: Inguinoescrotal hernia reduces with manual manipulation	F3: SF 25–34.9 mm	F3: BMI 30–34.9
H4: Irreductible inguinoescrotal Hernia	F4: SF > 35 mm	F4: BMI > a 35

*SF* scapular fold thickness, *BMI* body mass index

A previous study performed at Hospital del Mar [6] used a modification of this classification (KN) that replaced scapular fold thickness (SF) with the patient's body mass index (BMI) (Table 1).

In this study [6], from a sample of 256 patients, it was shown that scores greater than 5 required a longer operative time, but no significant relationship was demonstrated between the preoperative score and the complication rate, probably due to the small sample size. Differences in recurrences were also not detected. We hypothesized that the preoperative score could predict the development of postoperative complications in patients operated for primary unilateral inguinal hernia in a larger sample of patients.

The aim of this study is to analyse the postoperative complications (30 days) in patients undergoing primary inguinal hernia repair surgery and to test their correlation with the KN preoperative scoring system (Table 1).

The secondary aims are: to evaluate the correlation of KN score with recurrences and to study whether the combination of KN and postoperative (EHS) score increases the predictive capacity of complications.

## Methods

This is a prospective, observational, single-center study of patients operated for primary inguinal hernia at the Department of General Surgery of Hospital del Mar between 2019 and 2020. Study design was performed following STROBE [7] statements. Hospital del Mar is one of the centres participating in the Spanish Abdominal Wall Registry (EVEREG) [8]. Its main objective is to have a register of all surgical interventions related to the abdominal wall in Spain. Based on this information, the aim is to analyse the results of the surgeries and assess the rate of complications associated with the surgery, as well as to evaluate the different surgical techniques and associated materials.

Throughout the EVEREG National Inguinal Hernia Registry database, data related to patient demographics (age, weight, height, BMI, work activity, concomitant diseases, previous personal history of abdominal wall pathology), and related to hernia characteristics (time of evolution, accompanying symptoms, exploratory findings, classification according to modified Kingsnorth (KN) and EHS) were collected. Data regarding the surgical intervention, type of anaesthesia, surgical findings, management of the nerves of the inguinal region, operative time, intraoperative complications, days of hospital stay and whether the intervention was performed by a surgeon member of the Abdominal Wall Unit (AWU) were also recorded.

Inclusion criteria were: men aged > 18 years who underwent elective surgery for primary unilateral inguinal hernia and major outpatient surgery (MOS) or day surgery patients were also included. Exclusion criteria were recurrent hernias, bilateral hernias and female sex.

Follow-up was performed by physical exploration at 4 weeks, 6 months and 12 months after surgery to detect complications, recurrence and/or chronic pain and if doubt abdominal ultrasound or computed tomography scan by specialized radiologist as required were performed. Complications at 1 month were classified according to the Clavien–Dindo classification [9]. General complications during hospital stay and surgical wound-related complications (SSO) at 1 month, such as hematoma, seroma and surgical wound infection were analysed.

Complications and recurrence were analysed according to the KN score classification (2–8 points) and the EHS score classification (1–6 points), obtaining a combined classification ranging from 3 to 14 points (KN-EHS classification).

The sample size calculation was based on our previous experience [6] due to previous incidence of complications.

### Statistical analysis

The SPSS 25.0 program (IBM Inc. Rochester, MN, USA) was used for the statistical analysis. Quantitative variables were represented as mean and standard deviation (SD) or median with interquartile range (IQR). Qualitative variables were represented as proportions. The association between qualitative variables was analysed using contingency tables (Chi-square) and the odds ratio (OR) was calculated. The association between quantitative variables was analysed using Student’s t test for unpaired data. Statistical significance was established at *P* < 0.05.

Multivariate analysis was performed to identify risk factors for the development of complications. The predictive capacity of each variable and its independence from the other predictor variables were analysed using a binomial logistic regression model by sequentially entering the variables with an input F of 0.5.

Approval was obtained from the Clinical Research Ethics Committee (CEIC nº 2020/9578). The patients were informed, and the data were treated in accordance with Law 15/1999 on the Protection of Personal Data. The clinical trial protocol was registered under code NCT04806828 (ClinicalTrials.gov).

## Results

**Fig. 1:**
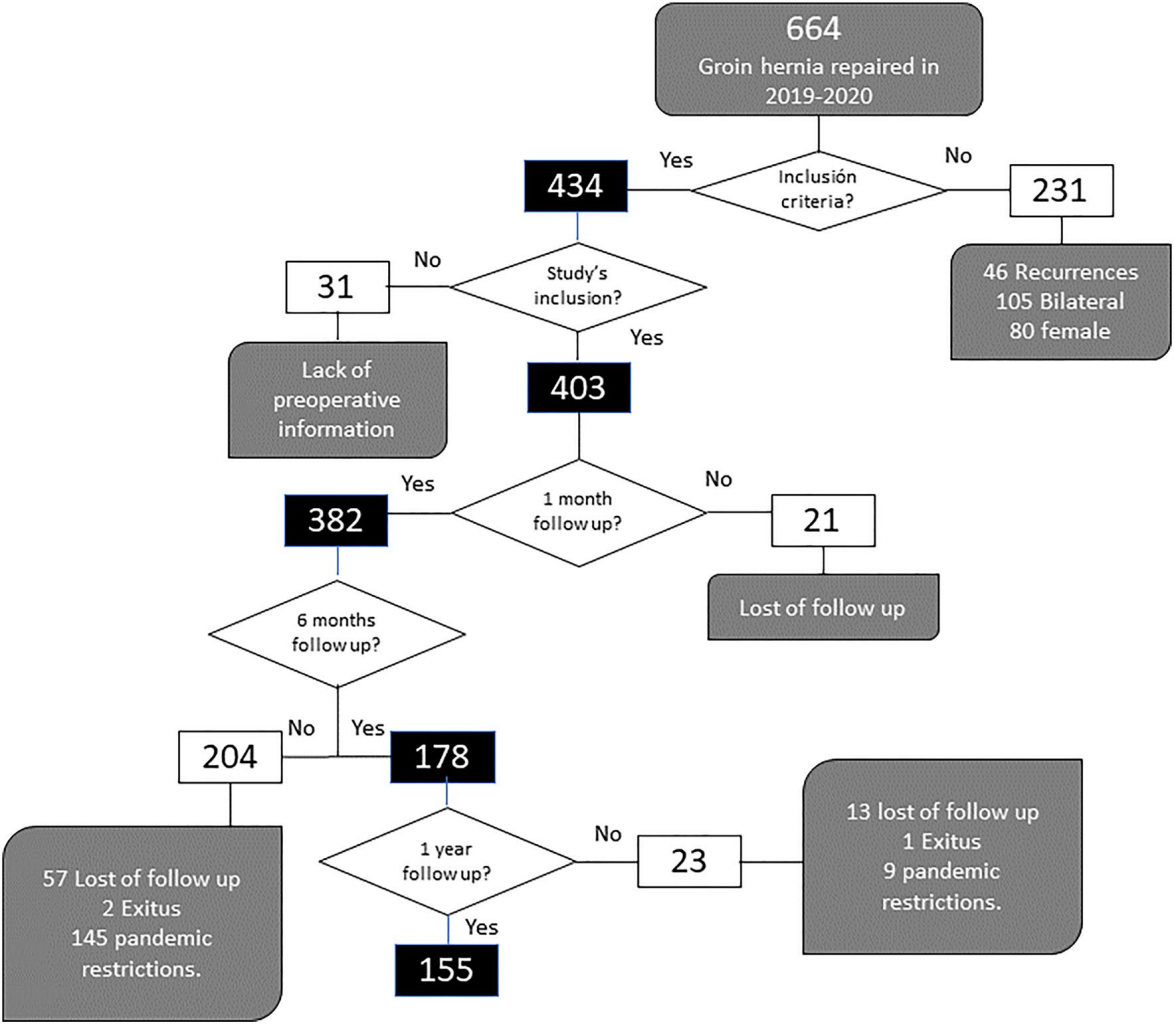
Flowchart. Hernias included in the study and follow-up at 1 month, 6 months and 1 year after the intervention

From January 2019 to December 2020, a total of 434 patients met the inclusion criteria; 31 patients were excluded due to lack of information on hernia classification, Fig. 1.

The demographic characteristics and pathological background of the patients, as well as preoperative characteristics of the patients' hernias and their scores are shown in Table 2. Hernias with KN 5–8 constituted 15.7% of the population.

**Table 2:** Patient demographics (*N* = 403)

Characteristics	*N* (%) o median (IQR)
Median age, years (IQR)	60 (50–72.1)
Age > 70 years, *N* (%)	120 (29.8)
BMI, median kg/m^2^ (IQR)	25.32 (23.5–27.7)
Smoking history, *N* (%)	178 (44.0)
Comorbidities, *N* (%)	173 (42.9)
Diabetes mellitus, *N* (%)	43 (10.7)
High blood pressure, *N* (%)	111 (27.5)
Cardiac disease, *N* (%)	40 (9.9)
Pulmonary disease, *N* (%)	48 (11.9)
Liver disease, *N* (%)	11(2.7)
Renal disease, *N* (%)	14 (3.5)
Stage IV renal disease, *N* (%)	1 (0.25)
Malignant neoplasia, *N* (%)	6 (1.5)
ASA classification III/IV, *N* (%)	61 (15.1)
KN 2, *N* (%)	101 (25.0)
KN 3, *N* (%)	134 (33.5)
KN 4, *N* (%)	105 (26.0)
KN 5, *N* (%)	39 (9.7)
KN 6, *N* (%)	20 (5.0)
KN 7, *N* (%)	4 (1.0)
KN 5–8, *N* (%)	63 (15.7)
KN-EHS 3–8, *N* (%)	280 (92.7)
KN-EHS 9–14, *N* (%)	22 (7.3)

*QR* interquartile range, *BMI* body mass index, *ASA* *American Society of Anesthesiologist*, *KN* modified Kingsnorth score, *KN-EHS* Kingsnorth score and EHS score addition

**Fig. 2:**
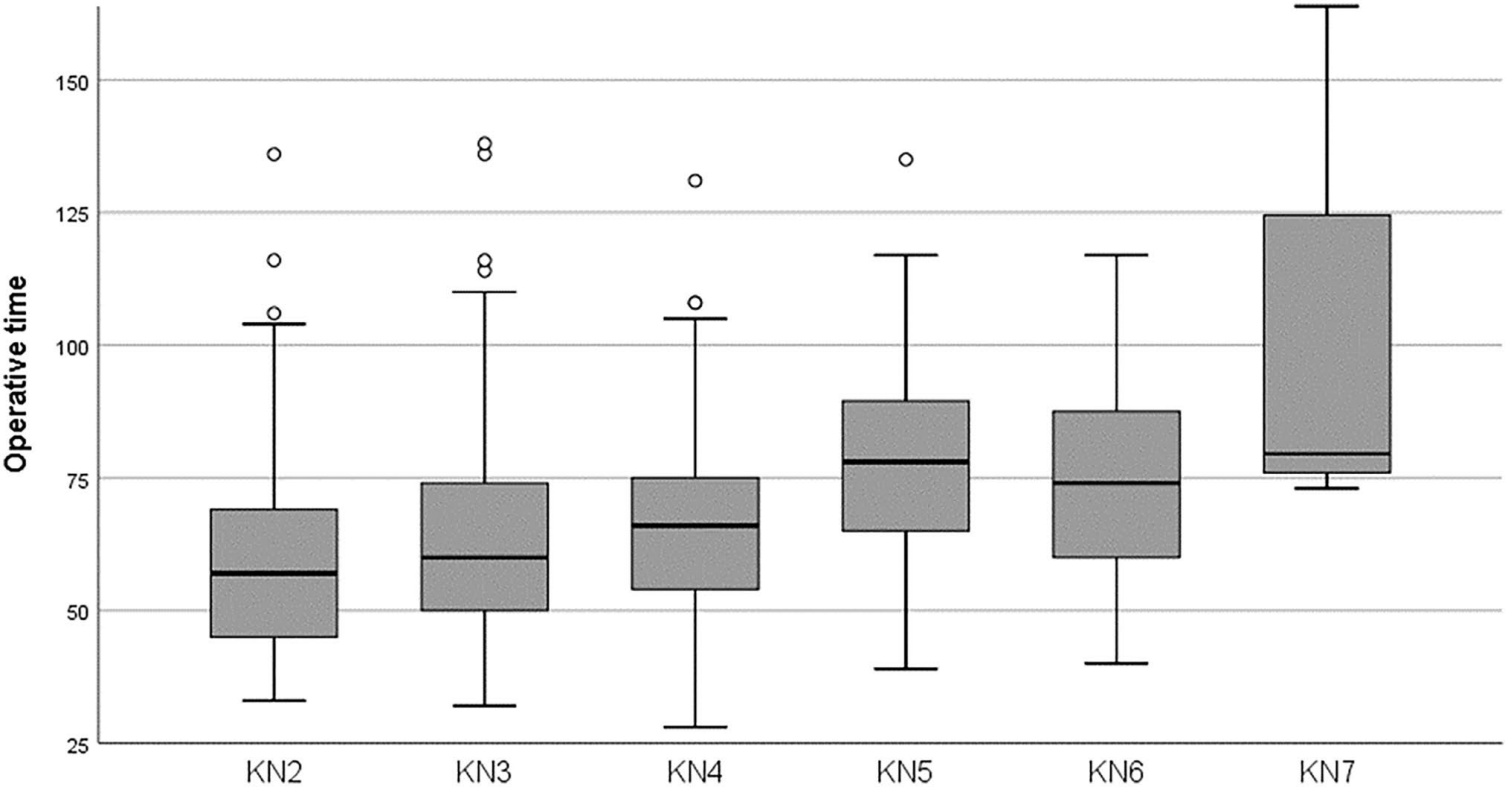
Operative time according to KN score

Table 3 shows the variables related to the operation. The mean operative time was 64 min. Most patients (61%) underwent major outpatient surgery (MOS). Figure 2 displays the analysis of the surgery duration related to the KN score. Higher KN score correlated with longer duration of surgery (Pearson correlation 0.291; *P* < 0.0001).

**Table 3:** Surgery-related variables

Variables	*N* (%)
Duration, median, min, (IQR)	64 (50–76)
MOS, *N* (%)	246 (61.0)
Mean overall hospital stay, days (SD)	0.51 (1.0)
Average MOS hospital stay, days (SD)	0.08 (0.3)
Operated by AWU surgeon, *N* (%)	85 (21.1)
General complications, *N* (%)	62 (15.4)
SSO, *N* (%)	40 (9.9)
Hematoma, *N* (%)	22 (5.5)
Seroma, *N* (%)	14 (3.5)
Surgical wound infection, *N* (%)	5 (1.2)
Clavien–Dindo	
Grade 0, *N* (%)	341 (84.6)
Grade I, *N* (%)	52 (12.9)
Grade II, *N* (%)	9 (2.2)
Grade IIIB, *N* (%)	1 (0.3)

*MOS* major outpatient surgery, *SSO* surgical wound-related complications, *AWU* abdominal wall unit

The overall complication rate (Table 3) was 15.4%, and the SSO rate was 9.9%. 83% of the complications were classified as Clavien–Dindo I.

**Table 4:** Univariate analysis of factors related to general complications and SSO

Variable (*N*)	General complications			SSO		
	*N*	OR (95% IC)	*P*	*N*	OR (95% IC)	*P*
Age > 70 years (120)	17	0.87 (0.48–1.6)	0.66	11	0.88 (0.43–1.83)	0.74
*Diabetes mellitus* (43)	10	1.8 (0.83–3.86)	0.13	7	1.92 (0.8–4.67)	0.14
ASA III/IV (61)	7	0.68 (0.29–1.57)	0.36	4	0.5 (0.21–1.74)	0.34
BMI > 30 (52)	11	1.58 (0.76–3.27)	0.21	7	1.49 (0.63–3.59	0.36
Smoking (178)	34	1.66 (0.96–2.86)	0.07	22	1.62 (0.84–3.13)	0.15
AWU surgeon (85)	6	0.36 (0.15–0.86)	0.02	3	0.28 (0.08–0.92)	0.03
KN 5–8 (63)	12	1.37 (0.68–2.74)	0.38	11	2.7 (1.07–4.82)	0.03
KN-EHS 9–14 (22)	4	1.33 (0.42–4.14)	0.62	4	2.61 (0.81–8.38)	0.10

*ASA* American Society of Anesthesiologists, *BMI* body mass index, *AWU* abdominal wall unit, *KN* modified KN score, *KN-EHS* sum of modified KN and EHS score

Analysis of complications according to KN score and KN-EHS score showed that KN5-8 scores had higher complication rate in comparison with lower scores. The KN-EHS score showed a similar distribution to the KN.

**Fig. 3:**
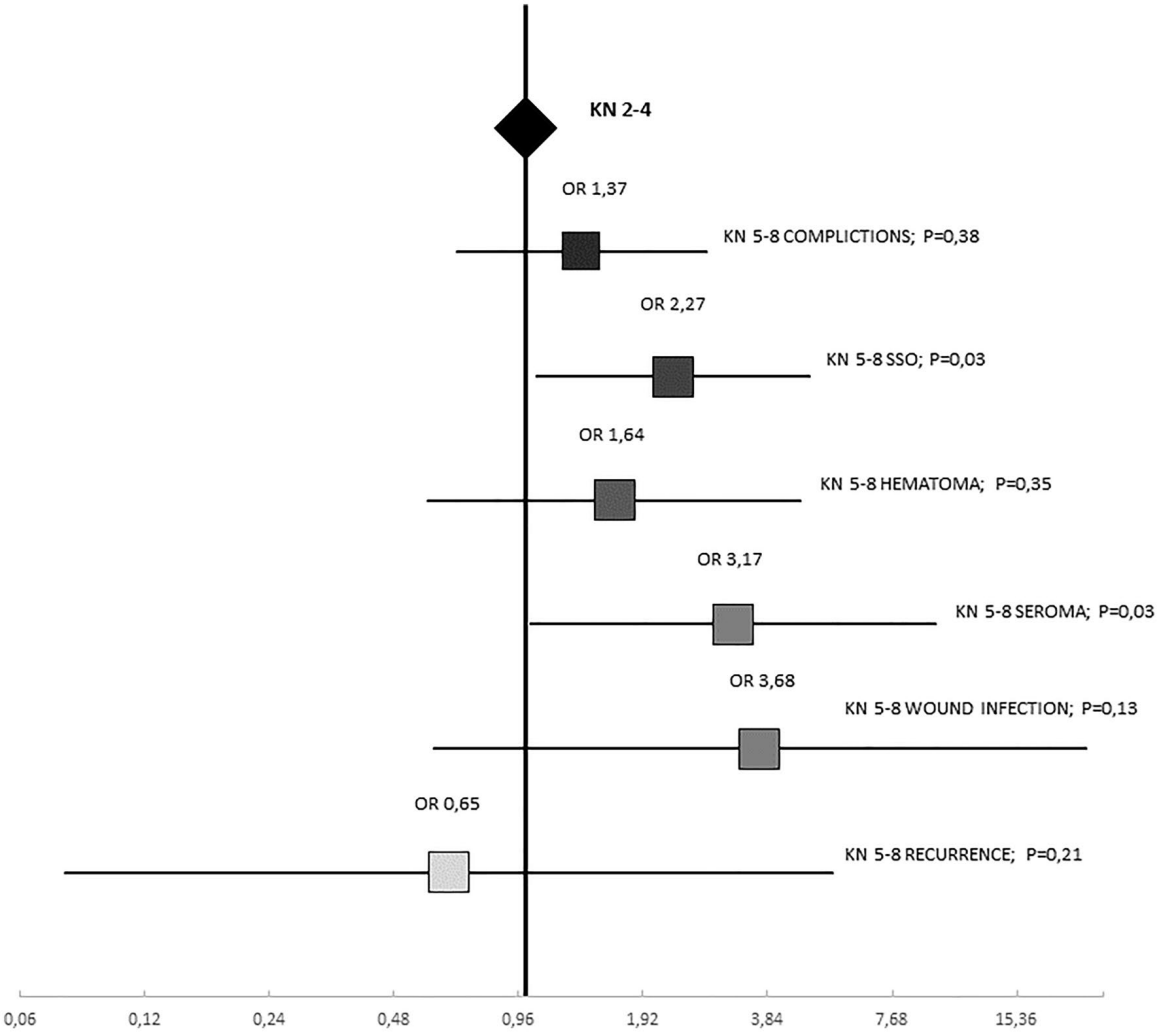
Graphical representation of the univariate analysis of the KN5-8 score and complications

When analysing the relationship of the study variables with the presence of overall complications and SSO (Table 4), significant differences were observed, presenting lower overall complications and SSO if the intervention was performed by an AWU surgeon. Significant results were also obtained relating the KN5-8 score to the presence of SSO. This result correlates with the results obtained in the detailed analysis of complications. The relationship between complications with the score is shown in Fig. 3. The sum of the two classification systems (KH-EHS 9–14) did not show statistically significant differences.

**Table 5:** Analysis of risk factors and complications for specialist abdominal wall surgeon (AWU) and non-specialist surgeon (non-AWU)

Variables	No AWU	AWU	P
Mean age, years (SD)	59.7 (14.4)	61.8 (16.1)	0.21
Age > 70 years, *N* (%)	87 (27.4)	33 (38.8)	0.04
Mean BMI, kg/m^2^ (SD)	25.6 (3.3)	26.0 (3.8)	0.04
Smoking history, *N* (%)	144 (45.3)	34 (40.0)	0.38
Comorbidities, *N* (%)	131 (41.2)	42 (49.4)	0.17
Diabetes mellitus, *N* (%)	33 (10.4)	10(11.8)	0.71
High blood pressure, *N* (%)	79 (24.8)	32 (37.6)	0.02
Cardiac disease, *N* (%)	29 (9.1)	11 (12.9)	0.29
Pulmonary disease, *N* (%)	41 (12.9)	7 (8.2)	0.24
Liver disease, *N* (%)	7 (2.2)	4 (4.7)	0.21
Renal disease, *N* (%)	10 (3.1)	4 (4.7)	0.48
Stage IV renal disease, *N* (%)	1(0.3)	0 (0.0)	0.60
Malignant neoplasia, *N* (%)	3 (0.9)	3 (3.5)	0.08
ASA III/IV, *N* (%)	46 (14.5)	15 (17.6)	0.47
KN 5–8, *N* (%)	43 (13.5)	20 (23.5)	0.02
KN-EHS 9–14, *N* (%)	13 (5.9)	9 (11.0)	0.13
Surgery duration, min (SD)	67.0 (19.8)	62.7 (29.8)	0.13
Total complications, *N* (%)	56 (17.6)	6 (7.1)	0.02
SSO, *N* (%)	37 (11.6)	3 (3.5)	0.03
Recurrences, *N* (%)	8 (7.0)	0 (0.0)	0.08

*BMI* body mass index, *ASA* American Society of Anesthesiologist, *KN* modified Kingsnorth score, *KN-EHS* sum of modified KN and EHS score, *SSO* surgical wound-related complications

In the binary logistic regression analysis, the fact that surgery was performed by a member of the AWU was presented as a protective factor for complications (HR 0.38; 95% CI 0.15–0.93; *P* = 0.035). When analyzing by the same method the incidence of SSO, two variables showed association with a lower frequency of complications: surgery performed by a member of the AWU (HR 0.24; 95% CI 0.07–0.82; *P* = 0.02) and KN score 2–4 points (HR 0.38; 95% CI 0.17–0.82; *P* = 0.01).

When analysing the composition of the cohort according to whether the operation was performed by a specialist surgeon (AWU) (Table 5), the AWU group had a significantly higher percentage of patients aged over 70 years, higher obesity rate and a higher frequency of KN score 5–8 patients.

Primary analysis was performed in 382 patients due to 21 patients did not perform 30-day postoperative outpatients clinical control. A total of 155 patients completed long-term follow-up with a mean follow-up of 38.8 months. Lost of follow-up was mainly cause lack of outpatient clinic control, others due to pandemic situation physical exploration was not possible. During follow-up, eight recurrences were diagnosed (5.1%). Univariate statistical analysis of recurrences demonstrated no significant differences related to KN score (OR 0.65; 95% CI 0.08–5.5; *P* = 0.69), nor to the operation performed by the AWU surgeon (OR 0.93; 95% CI 0.88–0.98; *P* = 0.08). The only significant risk factor for recurrences was a history of smoking (OR 8.91; 95% CI 1.07–74.22; *P* = 0.016).

## Discussion

Inguinal hernia classification is expected to unify criteria to describe accurately the anatomy of the inguinal canal defect [3, 4], and to provide a prediction model for outcomes after surgery. Our study is the first in the literature to correlate the preoperative classification of inguinal hernias with the development of complications and the duration of surgery.

The sample size calculation based on our previous experience [6] estimated a sampling of 630 patients. Due to the current situation caused by the COVID19 pandemic it has not been possible to reach the target as surgical activity has decreased for several months. Likewise, the operations during the year 2020 have been less complex, since priority was given to those that could be performed in a major outpatient surgery (MOS) regime or day surgery to avoid the patient's admission. All these factors have contributed to a lower percentage of patients with high scores than what was expected when designed.

The characteristics of the sample patients are similar to those published in the literature [10]. Regarding the KN classification, 85% of the patients presented scores that were related to low surgical complexity.

The variables related to the surgical procedure and its results were also comparable to those previously reported in other similar studies [8]. The operative time was shorter in patients with KN 2–4 without reaching statistically significant differences, most likely influenced by the sample size. However, Pearson's correlation showed an association between KN score and operative time. This is very interesting and has been previously reported [5, 6] and may be very useful to distribute operating room time in a cost-effective manner, allowing a greater or lesser number of patients to be scheduled depending on their score. Approximately each point of the KN classification correlates to 15 min of surgery, which allows us to calculate the time for surgeries between 30 and 120 min.

A low proportion of patients presented complications, most of them being mild (Grades I–II Clavien–Dindo [9]: 98.4% of all patients). Analysis of the variables related to postoperative complications failed to demonstrate a significant association with the overall KN score. Instead, wound-related complications showed a statistically significant relationship with KN score 5–8 (*P* = 0.03), at the expense of an increase in postoperative seromas. This confirms our initial hypothesis since wound complications are clearly influenced by the preoperative scoring system. This is important to provide more precise information to patients and to propose technical or therapeutic measures to prevent their occurrence. Surgical wound infection was not related statistically significant, but there is a clear trend towards a higher number of infections, which could indicate the need for preoperative antibiotic prophylaxis in patients with KN5-8 regardless of their risk factors [11].

The use of the summation of both scores (KN-EHS) did not provide greater accuracy for the prediction of complications.

An unexpected finding in our study was the statistically significant association of global and wound complications with operations performed by surgeons who were not members of the AWU. This is even more relevant if we examine the comparison of patients operated on by each group of surgeons (Table 5). AWU patients apparently had more risks factors for complications: older age, higher BMI and a higher percentage of patients with KN score 5–8 (AWU 23.5% vs. Non-AWU 13.5%; *P* = 0.02). In contrast, outcomes were better. This data suggest that inguinal hernia operations, especially cases with KN score 5–8, should be scheduled with surgeons specialized in abdominal wall surgery. Moreover, in the group classified as KN2-4, although statistical significance was not reached, there was a clear trend towards a lower frequency of global (OR 0.43; 95% CI 0.16–1.12; *P* = 0.08) and wound complications (OR 0.29; 95% CI 0.07–1.26; *P* = 0.08). Furthermore, it should be noticed that there were no wound infections or recurrence events in AWU-operated patients. Data shall be taken cautiously and confirmed with a prospective randomized control trial.

Regarding hernia recurrence, the overall rate was 5.1%, in accordance with those previously reported [2, 12]. In this case, the KN score did not demonstrate any benefit. Moreover, most recurrences appeared in patients with KN2-4 scores (87.5% of all recurrences). This has already been pointed out by other authors who detected that the frequency of recurrence in low complexity hernias was higher [13]. Likewise, it is noteworthy that there was no recurrence when the surgery was performed by an AWU surgeon. It should be taken into account that all operations in which a specialized surgeon was present, as surgeon or assistant, were considered as AWU operations. It seems that recurrences have a greater relationship with technique selection or operative events.

Subspecialisation in surgery has previously shown better results in other studies such as in colorectal surgery [14], oncology surgery [15]. Specifically in abdominal wall surgery, there are other studies on incisional hernia repair, where also better results had already been detected in operations performed by a specialized surgeon [16].

Another factor that appeared to be associated with recurrences was smoking history, both active and previous smokers. Smoking has been associated with a higher frequency of complications and recurrences in abdominal wall surgery previously [17–19]. In our study, it is remarkable that not only active smoking had an influence on recurrences, but a previous history of smoking addiction also had a negative impact on recurrences. This also deserves a prospective study with a larger sample size with patients without active smoking, but with a history of tobacco addiction to ensure a correct correlation with groin hernia recurrence.

The main strengths of this study are its prospective nature and the fact that data were collected in a unified national registry of inguinal hernia.

Weaknesses relate to the decreased sample size due to the current COVID-19 pandemic situation and the large amount of loss to follow-up due to cancellation of appointments for this same reason, so that only 44.2% of patients were followed long-term.

In conclusion, the KN classification allows predicting the occurrence of operative wound complications in patients operated on for unilateral primary inguinal hernia. KN score 5–8 has higher chances of wound complications. When surgery is performed by a specialist in abdominal wall surgery, chances of postoperative complication are lower.

## Data Availability

The datasets generated during and/or analysed during the current study are available from the corresponding author on reasonable request.

## Declarations

### Trial registration number

NCT04806828 (clinicaltrial.gov). Date of registration, March 19, 2020.
